# Development of Functional Biointerface Using Mixed Zwitterionic Silatranes

**DOI:** 10.1021/acs.langmuir.4c03302

**Published:** 2024-11-11

**Authors:** Thi Anh Hong Tran, Van Truc Vu, Chun-Jen Huang

**Affiliations:** a Department of Biomedical Sciences and Engineering, National Central University, Jhong-Li, Taoyuan 320, Taiwan; b Department of Chemical & Materials Engineering, National Central University, Jhong-Li, Taoyuan 320, Taiwan; c School of Materials Science and Engineering, The University of New South Wales, Sydney, NSW 2052, Australia

## Abstract

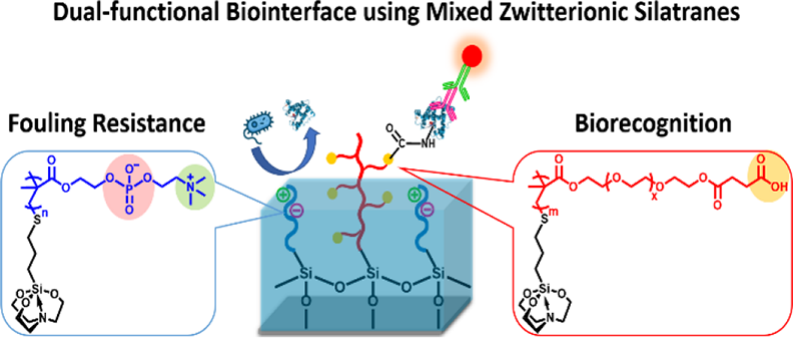

Strategies to design multifunctional interfaces for biosensors have been extensively investigated to acquire optimal sensitivity, specificity, and accuracy. However, heterogeneous ingredients in clinical samples inevitably generate background signals, exposing challenges in biosensor performance. Polymer coating has been recognized as a crucial method to functionalize biointerfaces by providing tailored properties that are essential for interacting with biological systems. Herein, we introduce for the first time two oligomeric silatranes, MPS–MPC_*n*_ and MPS–PEGMACOOH_*m*_, which were copolymerized from mercaptopropylsilatrane (MPS) with either zwitterionic monomer 2-methacryloyloxyethyl phosphorylcholine (MPC) or carboxylated poly(ethylene glycol) methacrylate (PEGMACOOH) through thiol–ene polymerization. These oligomeric silatranes were prepared individually and in combinations in acidic and nonacid solvents for deposition on silicon wafers. Afterward, coating properties, including wettability, thickness, and elemental composition, were characterized by contact angle meter, ellipsometer, and X-ray photoelectron spectroscopy (XPS), respectively. Importantly, MPS–MPC_*n*_ polymers were found to form thin films with high hydrophilicity and superior fouling repulsion to bacteria and protein, while mixed coating involving 70% MPS–PEGMACOOH_2.5_ and 30% MPS–MPC_2.5_ exhibited thinnest coating with best wettability among COOH-terminated coatings. Furthermore, the functional COOH group in the coated surfaces was exploited for postmodification with biological molecules via intermediated *N*-hydroxysuccinimide (NHS) ester group by amine coupling chemistry. Once again, the combination of 70% MPS–PEGMACOOH_2.5_ and 30% MPS–MPC_2.5_ provided an ultimate reduction in nonspecific adsorption (NSA) and established a finest signal discrimination through enzyme-linked immunosorbent assay. Consequently, these novel mixed oligomeric silatranes offer a promising approach for the construction of biosensor interfaces with dual functions in both nonspecific binding prevention and conjugation of biomolecules.

## Introduction

Biosensors are widely employed as analytical tools across agriculture, food safety, medical diagnosis, environmental remediation, and beyond, owing to their versatile surface functionalities, also known as the biointerface.^1^ Biointerface is decisive in key performance metrics of biosensors such as selectivity, sensitivity, detection range, reproducibility, stability, and biocompatibility. Thus, the performance of biosensor is challenged by factors that compromise the biointerface, including substrate defection, nonuniform immobilized layers, nonspecific adsorption, and aggregation phenomena.^2^ Surface modification strategies have been increasingly studied to address these challenges and offer optimized approaches for biointerface construction, emerging as self-assembled monolayers (SAMs) of carboxylated reagents, organosilanes, or alkanethiol derivatives; electrodeposition with fabricated metal nanoparticles; functionalized polymer coatings; diverse nanomaterials such as carbon nanotubes or graphene oxide; and metal–organic frameworks (MOF) as copper-based MOF and cerium MOF serve as active methods for mitigating these issues and optimizing biosensor performance.^3−6^ Importantly, nonspecific adsorption (NSA) of proteins, bacteria, and cells in the sample matrix can physically absorb on the biosensor surface via electrostatic forces, hydrogen bonding, and hydrophobic interactions. NSA results in elevated background signals, thereby affecting the limits of detection and decreasing the reproducibility, selectivity, and sensitivity. Consequently, minimizing NSA is crucial in the development of biosensors, particularly for implantable biosensors.^7−10^

Silane-based polymers have found widespread applications in hybrid thin films, cross-linking agents, sol–gel reactions, and anticorrosive coatings owing to their flexible features of thermal stability, chemical inertness, fouling repellency, low toxicity, and good adhesion to substates.^11−14^ However, their potential in biointerface functionalization has been limited due to difficulties in controlling the silanization process. The uncontrollable hydrolysis and accumulation of oligomers and polymers between silanol groups in a water-presented environment inevitably lead to the formation of thick disordered layers and big aggregations, reducing the uniformity and molecular orientation and diminishing the availabilities of functional end groups.^15,16^ Recently, silatrane was recognized as a promising approach to address the existing issues of the original silanes. Contrary to silane groups, silatranes possess a unique caged structure characterized by the tetrahedral arrangement with transannular N → Si dative bond that enhances their chemical stability and hydrolysis resistance and prevents self-polymerization. Therefore, not only inheriting the general advantages of organosilanes, silatranes also facilitate the controlled silanization to form thin, smooth, stable, and ordered SAMs. Compared with other surface modification methods, silanization involves the reaction of a silatranyl ring with hydroxylated surfaces to form a covalently bonded silane layer, offering recognized advantages such as durable and stable modifications, cost-effectiveness, simplicity, and versatility across various substrates including glass, metals, and ceramics. Furthermore, its scalability enhances its appeal. These strengths make silanization a compelling choice for applications in material science, biotechnology, and surface engineering, especially when stability, customization, and uniformity are critical.^17−20^ On the other hand, zwitterionic polymers (ZP) and poly(ethylene glycol) (PEG) are known for their excellent hydrophilicity, stability, biocompatibility, and antifouling behavior. Their ability to resist nonspecific protein adsorption and microbial attachment primarily relies on the combination of zwitterionic structure, hydration layer formation, steric hindrance, and dynamic surface properties.^21−26^ Notably, poly(sulfobetaine methacrylate) (PSBMA), poly(carboxybetaine methacrylate) (PCBMA), and poly(2-methacryloyloxyethyl phosphorylcholine) (PMPC) within the polybetaine group possess distinct functional groups, making them suitable for various applications such as antifouling properties, blood-contacted sensors, drug delivery, and surface coatings.^24,27−29^ Generally, each zwitterionic polymer provides different functional properties, making them suitable for various applications. Among these, our system combines PEGMA and MPC to leverage the strengths of both materials, leading to interfaces that are highly biocompatible, resistant to fouling, and capable of specific bioactivity. Accordingly, the integration of silatranes with any of these mentioned materials is worth investigating mean of constructing and functionalizing biointerfaces.^30^

**Scheme 1:**
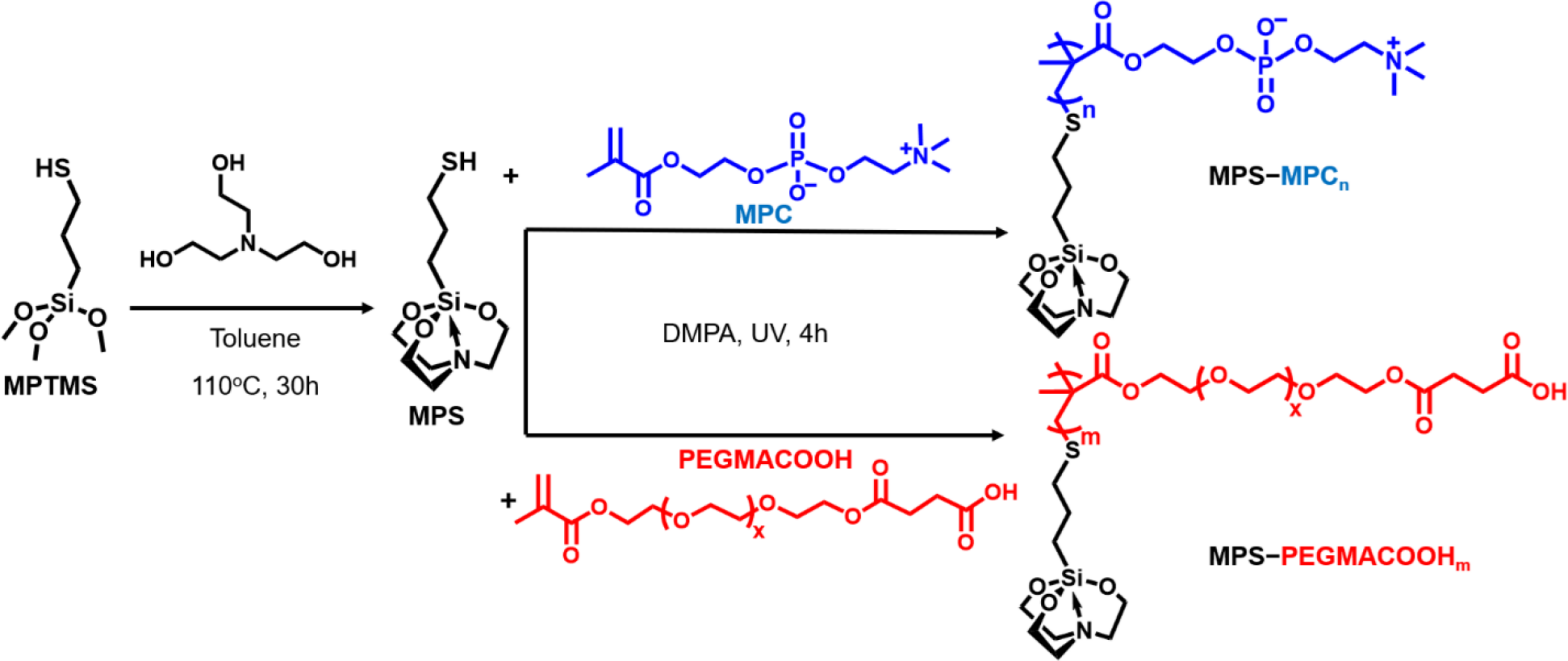
Synthesis Processes of MPS and Thiol–Ene Polymerization of MPS–MPC_*n*_ and MPS–PEGMACOOH_*m*_

**Scheme 2:**
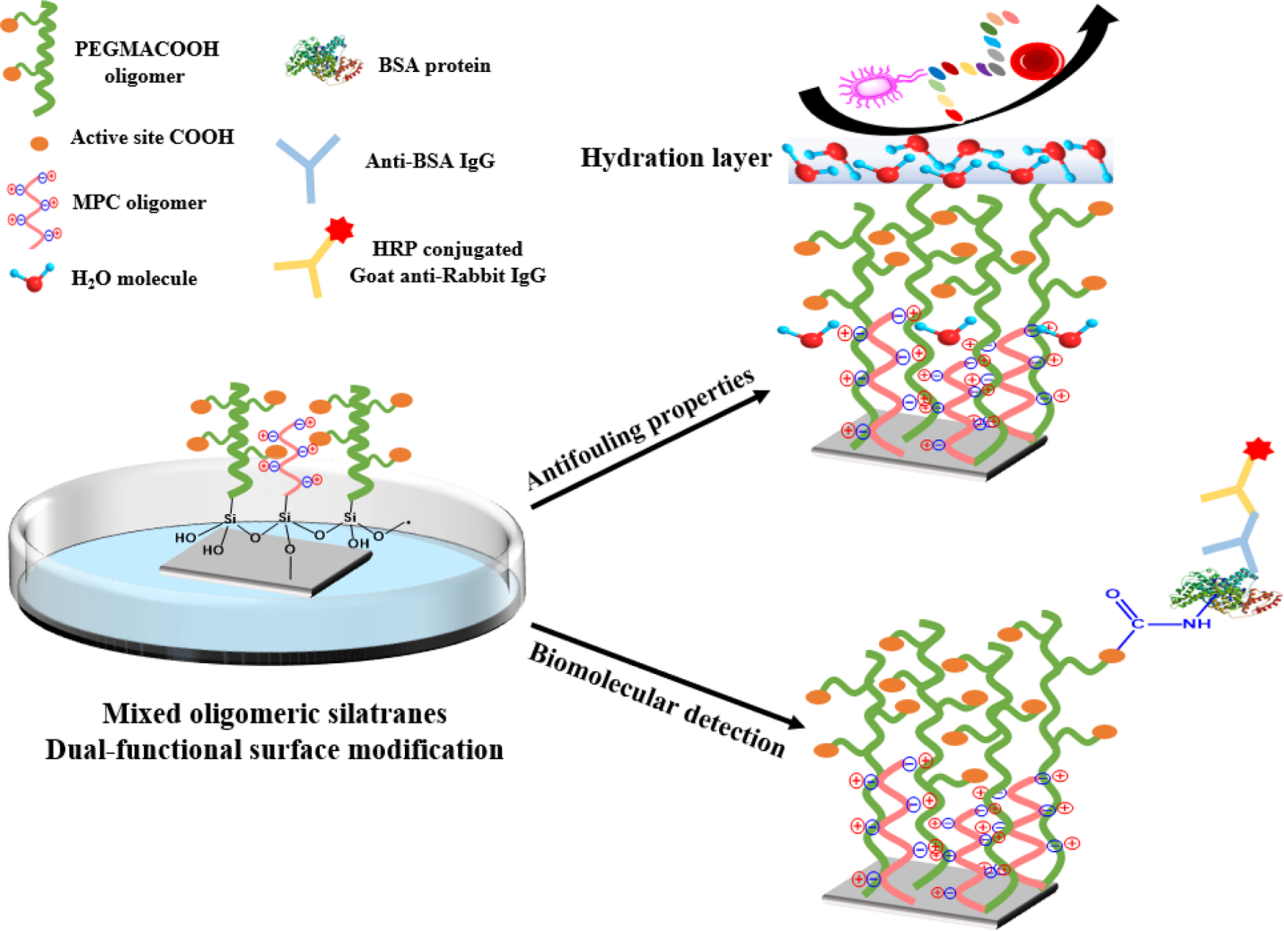
Dual-Functionality of Modified Mixed Oligomeric Silatranes on the Si Wafer

In this study, two oligomeric silatranes of MPS–MPC_*n*_ and MPS–PEGMACOOH_*m*_ were newly synthesized via thiol–ene polymerization under ultraviolet (UV) light across different feed ratios and 2,2-dimethoxy-2-phenylacetophenone (DMPA) photoinitiator, as summarized in Scheme 1. The chemical structure, conversion rate, and actual ratio of oligomeric silatranes were determined by using proton nuclear magnetic resonance (^1^H NMR) analysis. Subsequently, MPS–MPC_*n*_ and MPS–PEGMACOOH_*m*_ were prepared in anhydrous methanol containing 2% (v/v) acetic acid and deposited on the Si wafer surface to form thin films via a controlled silanization process. Worth mentioning, the mixed coatings of MPS–MPC_*n*_ and MPS–PEGMACOOH_*m*_ were studied to integrate their advantages and establish dual functional interfaces, as illustrated in Scheme 2, that not only effectively resist nonspecific adhesions but also facilitate the accessibility to functional COOH groups. Surface characterization including wettability, thickness, and elemental compositions was assessed by a water contact angle goniometer, an ellipsometer, and an XPS instrument, respectively. Moreover, a comprehensive comparison of MPS–MPC_1_, MPS–MPC_2.5_, and MPS–MPC_5_ interfaces in terms of antifouling performance was conducted, involving Gram-positive and Gram-negative bacteria and BSA (bovine serum albumin) protein. Ultimately, mixed oligomeric silatranes of MPS–MPC_*n*_ and MPS–PEGMACOOH_*m*_ were investigated the bioconjugation competence with the BSA protein model via colorimetric enzyme-linked immunosorbent assay (ELISA).

## Materials and Methods

### Materials

Bovine serum albumin (BSA, ≥ 98%), succinic anhydride (≥99%), poly(ethylene glycol)methacrylate (average M.W. = 500 Da), 2-methacryloyloxyethyl phosphorylcholine (MPC, 97%), and acetic acid (≥99%) were purchased from Sigma-Aldrich. Toluene (HPLC), 2,2-dimethoxy-2-phenylacetophenone (DMPA, 99%), 3-mercaptopropyl trimethoxysilane (MPTMS, 95%), 1-ethyl-3-(3-dimethylaminopropyl) carbodiimide (EDC, 97%), *N*-hydroxysuccinimide (NHS, 98%), triethanolamine (TEOA, 99+%), dichloromethane (DCM, 99.8%), diethyl ether anhydrous (DE, 99+%), chloroform (CHCl_3_, 99+%), dimethyl sulfoxide (DMSO, GC), horseradish peroxidase (HRP)-conjugated secondary antibodies, and rabbit anti-BSA IgG were acquired from Thermo Fisher Scientific. *N*-Pentane (99%) was supplied by Pharmco Aaper. The LIVE/DEAD BacLight Bacterial Viability kit was obtained from Life Technologies. Luria–Bertani (LB) agar was bought from BD (NJ). *Staphylococcus epidermidis* (*S. epidermidis*) and *Escherichia coli* (*E. coli*) were provided by the Bioresource Collection and Research Center of Taiwan. All other chemicals were analytical-grade.

### Synthesis and Characterization Mercaptopropyl Silatrane (MPS)

Previously described,^31^ MPTMS (3.3 mL, 18 mmol), TEOA (2.83 mL, 18 mmol), and NaOH (5 mg) as a catalyst were dissolved in 30 mL of toluene. The mixture was stirred in a two-neck round-bottom flask and refluxed at 110 °C for 30 h. After reaction, the system was cooled at room temperature (RT) and solvent was removed using a rotary evaporator. The concentrated solution was added dropwise to 200 mL of *n*-pentane for precipitation, and a light yellow solid of MPS was collected after filtration. Ultimately, vacuum-dried MPS powder should be stored at 4 °C as an initial powder form or stock solution of 460 mM in DMSO solvent. The molecular structure of MPS was verified using proton nuclear magnetic resonance (^1^H NMR) spectrometry (600 MHz, CDCl_3_).

### Synthesis and Characterization of Carboxylated-PEGMA

Poly(ethylene glycol) methacrylate (5 g, 10 mmol) and succinic anhydride (1 g, 10 mmol) were dissolved in a round-bottom flask containing 100 mL of chloroform as the solvent, along with a small amount of triethylamine as a catalyst. The system was then connected to a serpentine condenser and stirred at 70 °C for 24 h. Upon reaction, the mixture was washed twice with deionized (DI) water before the solvent was evaporated in a vacuum rotavap. The resulted brown liquid was later undergone a silica gel column chromatography system eluted by a mobile phase of gradient toluene–ethyl acetate–methanol to gain purified carboxylated PEGMA. The final product was stored at RT and characterized by attenuated total reflectance-Fourier transform infrared (ATR-FTIR) spectrometry, ^1^H NMR, and ^13^C NMR (600 MHz, MeOD).

### Synthesis of the Oligomeric Silatranes MPS–MPC_*n*_ and MPS–PEGMACOOH_*m*_

Thiol–ene polymerization involves three main steps: initiation, propagation, and termination. The reaction typically begins with the generation of radicals using thermal initiators (e.g., azo compounds) or photoinitiators that produce radicals upon exposure to UV light. The radical reacts with an alkene, creating a thiyl radical. In the propagation step, the thiyl radical adds to the double bond of an alkene, forming an alkyl radical. The new radical then reacts with another thiol or alkene, continuing the propagation cycle and promoting the growth of the polymer chain. Termination can occur through mechanisms such as the combination of two radicals or disproportionation, where one radical abstracts a hydrogen from another. The reaction can also be intentionally terminated by introducing additional thiol groups, which leads to cross-linking and the formation of network structures.^32,33^ In this study, 3.25 mL of MPS stock solution in DMSO (1.5 mmol) was mixed with either MPC or PEGMACOOH at the desired molar ratio in anhydrous methanol for the thiol–ene reaction, triggered by 2,2-dimethoxy-2-phenylacetophenone (DMPA). The reaction was conducted within nitrogen conditions and placed upon UV illumination at 365 nm in 4 h. After polymerization, the solvent was eliminated by a rotary evaporator to collect a concentrated solution that was later precipitated in a mixed solvent of ethyl ether and dichloromethane in a 2:1 ratio. The obtained oligomeric silatranes were purified with ethyl ether and finally dried under a vacuum line for 4 °C storage and further experiments.

### Surface Silanization with Oligomeric Silatranes

Silicon wafer was cut into small pieces of 1 × 1 cm^2^ and pretreated with a piranha solution to clean organic residues off substrates. After 40 min of immersion, these substrates were successively ultrasonicated in deionized water, ethanol, and acetone for 10 min each time. The cleaned substrates were dried with nitrogen steam and treated in oxygen plasma for 15 min to remove the remaining contamination and create the hydroxyl group on the surfaces. OH-terminated substrates were immediately immersed in the prepared coating solutions containing 5 mM MPS–MPC_*n*_, 5 mM MPS–PEGMACOOH_*m*_, or a 5 mM mixture in varying molar fractions. The coating solutions were previously prepared in anhydrous methanol with and without 2% (v/v) acetic acid. This silanization process occurred at 40 °C in 4 h for the deposition of silatranes onto the surfaces. Afterward, the solutions were withdrawn while substrates were sonicated in methanol for 10 min and dried by a nitrogen flow to eliminate unbound materials. Finally, all samples were placed in an oven at 60 °C for 1 h to produce a stable formation of the Si–O–Si covalent bonding. The modified samples can be stored in a clean and dry environment for the measurements.

### Attenuated Total Reflectance-Fourier Transform Infrared (ATR-FTIR)

ATR-FTIR is utilized for identifying functional groups and chemical compositions of synthesized materials through their infrared spectra ranging from 4000 to 650 cm^–1^. The procedure involves scanning the environmental background before placing samples onto the detector area, which was conducted by a Fourier transform infrared spectrometer (Bruker, Vertex 80v, Germany).

### Water Contact Angle Measurement

The solid wettability of modified surfaces was determined via the static contact angles, recorded by a contact angle meter (Phoenix mini, Surface Electro Optics, Seoul, Korea). Five microliters of deionized water was dropped from the syringe onto at least three different positions on each surface, and the contact angle formed at the interface due to the force balance of the three-phase system (solid–liquid–gas) of water droplet was measured.

### Thickness Measurement

An ellipsometer with three incident angles of 65, 70, and 75° was involved to calculate the film thickness on three separate locations. The data were fitted using a combined model of Si with thermal oxide and cauchy to accurately determine the thickness of the self-assembled layer on the surface. The coating thickness is ascertained as the difference between the thickness at the same location of the wafers before and after the silanization process.

### X-ray Photoelectron Spectroscopy (XPS) Characterization

XPS machine equipped with a microfocused and monochromatic Al Kα X-ray source is widely used for surface analysis to investigate the chemical composition of modified substrates. The substrate analysis was performed under a high vacuum pressure of 10^–8^ Pa along with a takeoff angle of the photoelectron of 23.5 eV. The photoelectron spectra were acquired with a constant analyzer pass energy of 23.5 eV. The resolution was maintained within 0.2 eV and calibrated against the Si 2p_3/2_ peak at 99.4 eV. Spectra of elements C 1s, N 1s, and P 2p were calibrated by using the characteristic peak of C 1s is 284.8 eV. Data was analyzed using a Gaussian function in Origin software.

### Bacteria Adhesion Test

Ten microliters of each *Escherichia coli* (Gram-negative) and *Staphylococcus epidermidis* (Gram-positive) were separately inoculated into tubes containing 10 mL of Luria–Bertani (LB) liquid medium and shaken at 150 rpm for 16 h overnight in a sterilized incubator at 37 °C with 5% carbon dioxide and 95% air, typically optimum conditions for cell culture. Following this, the optical density (OD) values at 600 nm were measured. The bacterial solutions were then centrifuged at 9000 rpm for 5 min, and the supernatant was dismissed. The precipitate *E. coli* and *S. epidermidis* were resuspended in 19.2 and 19.3 mL of PBS 1× buffer, respectively, and diluted to a fixed concentration corresponding to an OD value of 0.1. Bare and coated substrates were washed with sterilized 2 mL PBS 1× before incubation in bacterial solutions at the same conditions for 3 h. Thereafter, the bacterial solution was removed, and the wafers were shaken at 100 rpm in 5 min with 2 mL PBS 1× for the removal of unattached organisms. This step was repeated three times, and the absorbed bacteria on the surfaces were subsequently stained with fluorescent dye Live-Death BacLight at RT for 15 min. The substrates were then cleaned with 2 mL of PBS 1× for 5 min and transferred to a new 24-well plate. Lastly, the surfaces were observed under a fluorescent microscopy (Zeiss Microscope Axio Observer A1, Germany), and five different positions of each sample were randomly captured. The images were analyzed using ImageJ software to count the number of attached bacteria.

### Protein Adsorption Test

BSA is a representative protein tested for the antifouling ability of polymeric coatings through the ELISA method based on the specific antigen–antibody interaction. Earlier, purchased BSA was dissolved in 1× PBS to a concentration of 4.5 mg/mL while rabbit anti-BSA IgG (primary antibody) and goat antirabbit IgG antibody (HRP-conjugated secondary antibody) were both diluted to a concentration of 5.5 μg/mL in PBST (PBS containing 0.05 wt % Tween 80). The modified substrates were individually placed in a 24-well plate, and 1 mL of the prepared BSA solution was added to each well so that the liquid covers the entire surface. The plate was immediately placed in an oven at 37 °C and gently shaken at 80 rpm. After 3 h of incubation, the BSA solution in all the wells was removed, and all samples were washed by 5 min shaking at 80 rpm with 1 mL of PBST. This step was repeated three times to wash away disengaged BSA. Next, the substrates were immersed in prepared primary antibody solution and kept incubating for 1 h under similar conditions for BSA. Following, liquid was removed and a similar washing step was performed three times for 15 min. The subsequent step is dipping all substrates into prepared secondary antibody for the secondary antibody to link with the primary antibody for 1 h under a previously used condition. The samples were placed in a clean 24-well plate after last washing steps, and 600 μL of 3,3′,5,5′-tetramethylbenzidine (TMB) was added to each well. After 10 min, the reaction was stopped by adding 400 μL of H_2_SO_4_ 1 M (TMB:H_2_SO_4_ = 3:2). Eventually, yellow solutions with varying degrees of intensity were formed, and 300 μL of each was transferred to a fresh well of a 96-well plate for colorimetric quantification at OD_450_.

### Activation Process of Available Carboxyl End Groups

To start with, the COOH-terminated substrates were placed into a 24-well plate and a 15 mL mixture of EDC:NHS (4:1) was prepared in 0.1 M 2-(*N*-morpholino) ethanesulfonic acid (MES) buffer at pH 6. Next, 0.5 mL of the above EDC/NHS solution was added to each well to stimulate active site carboxylic acid on the surface. This activation process was carried out at RT for 1 h. Subsequently, the samples were washed three times with MES buffer to remove unreacted materials.

### Influence of Varied pH Conditions on BSA Immobilization

BSA has an isoelectric point (IEP) between pH 4.5 and 5.0 and, therefore, exhibits a negative charge at neutral pH.^34^ In this study, BSA was captured by activated available carboxylic acid on MPS–PEGMACOOH_*m*_ of the modified surface through NHS-ester to form a covalent binding. This process was performed across 3 pH values: pH 2, pH 5, and pH 7.5. The capability of BSA immobilization onto the surface was assessed using the ELISA method.

### BSA Immobilization on the Functionalized Interface

Prior to experiment, BSA was serially diluted in PBS buffer at pH 7.5 with several concentrations ranging from 25 to 1000 μg/mL. After the activation process, Si wafers coated by mixed oligomeric silatranes of 70% MPS–PEGMACOOH_2.5_ and 30% MPS–MPC_2.5_ were soaked in ready BSA solutions for 4 h at 37 °C for BSA protein to be apprehended. The samples were then rinsed three times with 1 mL of PBST to remove the unattached BSA, followed by 60 min immersion in 1 mL of ethanolamine in borate buffer at pH 8.5 at RT to block the remaining NHS-ester groups. The modified substrates were sequentially immersed in 1 mL of anti-BSA IgG 5.5 μg/mL and 1 mL of goat antirabbit IgG antibody (5.5 μg/mL). Each antibody solution was incubated for 1 h and followed by rinsing with PBS 1×. Subsequently, the substrates were subjected to 600 μL of TMB colorimetric indicator, and the reaction was halted by 400 μL of 1 M H_2_SO_4_ 10 min later. Finally, a 96-well plate containing 300 μL of each final solution was read with an ELISA plate reader at OD_450_.

### Antibody Detection on the Functionalized Surface

A primary antibody anti-BSA IgG solution prepared in sodium carbonate buffer at pH 8.5 at a constant concentration of 11 μg/mL was added (1 mL) onto each coated sample and incubated at 37 °C for 1 h for primary antibodies to stably conjugate with COOH groups through covalent bonding. Afterward, 1 mL of ethanolamine (1 M) in borate buffer was utilized to deactivate the free NHS-ester groups for 1 h at RT. Subsequently, triple wash with 1 mL of PBS buffer was performed and proceeded to incubate the substrates with HRP conjugated goat antirabbit IgG secondary antibody solutions at different concentrations of 5.5, 11, 16.5, 22, and 27.5 μg/mL for 1 h at 37 °C. After another triple wash with PBS buffer, total 1 mL of TMB and H_2_SO_4_ in a ratio of 3:2 was sequentially dispensed to each wafer, resulting in the yellow solutions of different intensities of which absorbances were measured at OD 450 nm.

### Statistical Analysis

Statistical analyses were performed using Student’s *t* test to identify among different testing factors. *P* value of less than 5 was considered as a statistically significant difference. Both data analysis and result presentation were performed with Origin 8.5.1 software.

## Results and Discussion

### Synthesis and Characterization of Molecular Building Blocks

The molecular building blocks of MPS and carboxylated PEGMA (PEGMACOOH) were synthesized for thiol–ene polymerization, as detailed in the Materials and Methods section. Briefly, MPTMS was reacted with TEOA refluxed at 110 °C in 30 h. The ^1^H NMR spectrum (600 MHz, CDCl_3_) of MPS is depicted in Figure S4a in which the proton distribution are illustrated at: δ (ppm) = 0.285 (2H), 1.22 (1H), 1.55 (2H), 2.64 (2H), 2.788 (6H), and 3.647 (6H). Most notable are two prominent peaks that present the tricyclic mercaptonitrosilyl ring of silatrane structure (OCH_2_, 6H and NCH_2_, 6H) at 2.788 and 3.647 ppm, proving that the synthesis was successful. Other necessary precursors for the thiol–ene polymerization to oligomeric silatranes are commercially available MPC and synthesized PEGMACOOH. PEGMACOOH as a brown liquid product was obtained from the reaction between succinic anhydride and hydroxyl-terminated PEGMA (PEGMAOH) after purification through silica gel column chromatography. Its characterization, including ^1^H NMR, ^13^C NMR, and ATR-FTIR spectra, is presented in Figures S1–S3, respectively. The synthesis of MPS and PEGMACOOH yielded approximately 80.9 and 70.5%, respectively.

Oligomeric silatranes debuted in this study, MPS–MPC_*n*_ and MPS–PEGMACOOH_*m*_, were harvested via thiol–ene polymerization with a photoinitiator DMPA in a nitrogen atmosphere under UV light for 4 h. Three oligomeric silatranes formed from MPS and MPC (MPS–MPC_1_, MPS–MPC_2.5_, and MPS–MPC_5_) were prepared in three feed ratios of 1:1, 1:2.5, and 1:5, respectively, whereas two feed ratios 1:2.5 and 1:5 of MPS:PEGMACOOH were similarly applied to generate the corresponding MPS–PEGMACOOH_2.5_ and MPS–PEGMACOOH_5_. General representative spectra of both MPS–MPC_*n*_ and MPS–PEGMACOOH_*m*_ characterized by ^1^H NMR (MeOD, 600 MHz) are shown in Figure S4b,c, while the detailed spectra of MPS–MPC_*n*_ and MPS–PEGMACOOH_*m*_ with different feed ratios are provided in Figures S5 and S6, respectively. As disclosed in Figure S4b, the distinguished peaks at 3.20–3.49 ppm (f, CH_3_, 9H) and 4.14–4.41 (d, CH_2_, 2H) represent the methyl protons attached to the quaternary ammonium group and methylene protons attached to the phosphoryl group, respectively. Besides, the peaks appear at chemical shifts δ of 3.62–3.79 ppm (e, CH_2,_ 2H) and 4.00–4.14 ppm (c, CH_2_, 2H) demonstrated that MPC successfully conjugated with MPS via the thiol–ene reaction. On the other hand, PEGMACOOH was effectively grafted to MPS for formation of the free-radical-mediated S–C bond, which is confirmed by the ^1^H NMR spectrum of MPS–PEGMACOOH_*m*_ in Figure S4c via the conspicuous peaks occur at 2.54–2.42 ppm (a, (CH_2_)_2_, 4H), 3.54–3.79 ppm (d,e (CH_2_)_2_, 4H), and 4.10–4.21 ppm (g, (CH_2_)_2_, 4H). Moreover, the molar ratio, conversion rate, degree polymerization, and yield of two oligomeric silatranes were determined from the ^1^H NMR spectra and are listed in Table 1. It is noteworthy that the actual ratios of silatrane and zwitterionic polymers in MPS–MPC_*n*_ and MPS–PEGMACOOH_*m*_ closely resemble the reaction ratios. The feed ratio (the ratio of monomers introduced into the reaction) and the actual polymer composition (the ratio of monomers in the final polymer) can differ for several reasons. Each monomer has a specific reactivity toward itself and the other monomer, meaning that polymerization may proceed in segments where one monomer dominates early on due to faster kinetics, resulting in a final composition that does not match the feed ratio. Additionally, the molecular weight of the initial monomers and the produced polymers can influence the polymerization kinetics. Furthermore, chain transfer and termination events can alter the final composition, as the likelihood of chain propagation versus termination changes as the polymer chains grow.^35−38^ Nevertheless, the conversion rate, or the percentage of monomers consumed in forming a polymer chain, exceeds 87% for oligomeric silatrane MPS–MPC_*n*_ and 81% for MPS–PEGMACOOH_*m*_, which indicates the efficiency of thiol–ene polymerization between the thiol group on silatrane and the methylene group in MPC and PEGMACOOH. In addition, the yield of each oligomeric silatrane was achieved in a range of 37.9–56.1%, depending on the molar ratios of each oligomeric silatrane. The degree of polymerization (DP) presents the number of monomer MPC_*n*_ and PEGMACOOH_*m*_ units in the oligomeric silatrane, which were calculated from the integral values of methylene peak at 0.28–0.38 ppm (SiCH_2_CH_2_) of MPS with a peak at 4.00–4.14 ppm (OCH_2_) of MPC and with a signal peak at 4.10–4.21 ppm (O(CH2)2O(CO)) of PEGMACOOH_*m*_ in the ^1^H NMR spectrum in Figures S5 and S6.^39^

**Table 1:** Parameters Analyzed Based on NMR of Oligomeric Silatrane MPS–MPC_*n*_ and MPS–PEGMACOOH_*m*_

**sample**	**feed ratio**	**actual ratio**	**conversion rate (%)**	**DP (degree polymerization)**	**crude yield (%)**
MPS–MPC_*n*_	1:5	1:5.38	87.8	8.3	37.9
	1:2.5	1:2.44	90.5	4.5	47.5
	1:1	1:1.11	96.5	2.3	55.3
MPS–PEGMA COOH_*m*_	1:5	1:4.77	81.6	5.9	47.5
	1:2.5	1:2.81	90.5	4.0	56.1

### Surface Modification and Characterization with MPS–MPC_*n*_ on Si Wafers

**Figure 1:**
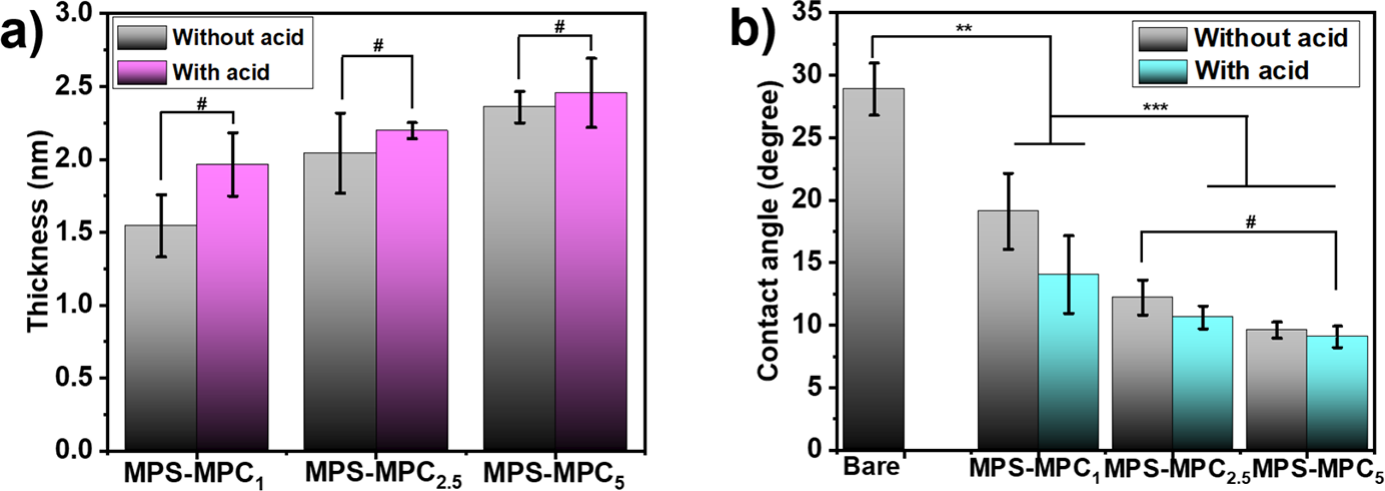
(a) Thicknesses and (b) static water contact angles of MPS–MPC_*n*_ coatings in solvents with and without acetic acid (*n* = 3, #*p* > 0.05, *p ≤ 0.05, **p ≤ 0.01, ***p < 0.001).

Thickness is always valuable information when analyzing the coating properties and effectiveness. In this study, an ellipsometer was employed to determine layer thicknesses of MPS–MPC_*n*_ via polarized light and the results are displayed in Figure 1a. After 4 h of deposition in an acidic environment, the thicknesses of MPS–MPC_1_, MPS–MPC_2.5,_ and MPS–MPC_5_ films were 1.97 ± 0.22, 2.20 ± 0.06, and 2.46 ± 0.24 nm, respectively, which show no significant difference compared to the films obtained without acid. Acetic acid has previously been shown to accelerate the hydrolysis of organosilanes, thereby enhancing the silanization process.^17^ As a result, the formation of oligomeric silatrane MPS–MPC_*n*_ films in an acidic solvent is not only more efficient but can also be completed in a shorter time. Interestingly, MPS–MPC_1_, with a degree of polymerization of 2.34, produced the thinnest film among the three polymers, and the film thickness increased with the MPC ratio. This suggests that oligomeric silatranes with longer chain lengths result in thicker films.

Zwitterionic polymers provide strongly antifouling properties due to their high hydrophilicity. Thus, the contact angle formed between a water droplet and tested surface was measured via a contact angle goniometer to assess the hydrophilicity of a bare silicon wafer and modified surfaces. As depicted in Figure 1b and Figure S7, the MPS–MPC_*n*_ surfaces exposed good wettability, evidenced by low contact angles of 19.1 ± 3° without acid and 14 ± 3° with acid addition compared to the bare Si wafer (28.9 ± 2°). This was to be expected since MPC is a well-known zwitterionic material with a superhydrophilic property. It can be observed that WCA of the samples coated with MPS–MPC_*n*_ in a solution with 2% acetic acid was slightly lower than those without acid. Generally, MPS–MPC_5_ with the highest polymer composition exhibited the longest polymer chain length and the lowest contact angle value. This outcome is attributed to the abundant MPC units grafted onto the oligomeric silatrane.

### Antifouling Properties of MPS–MPC_*n*_ Films on Modified Substrates

**Figure 2:**
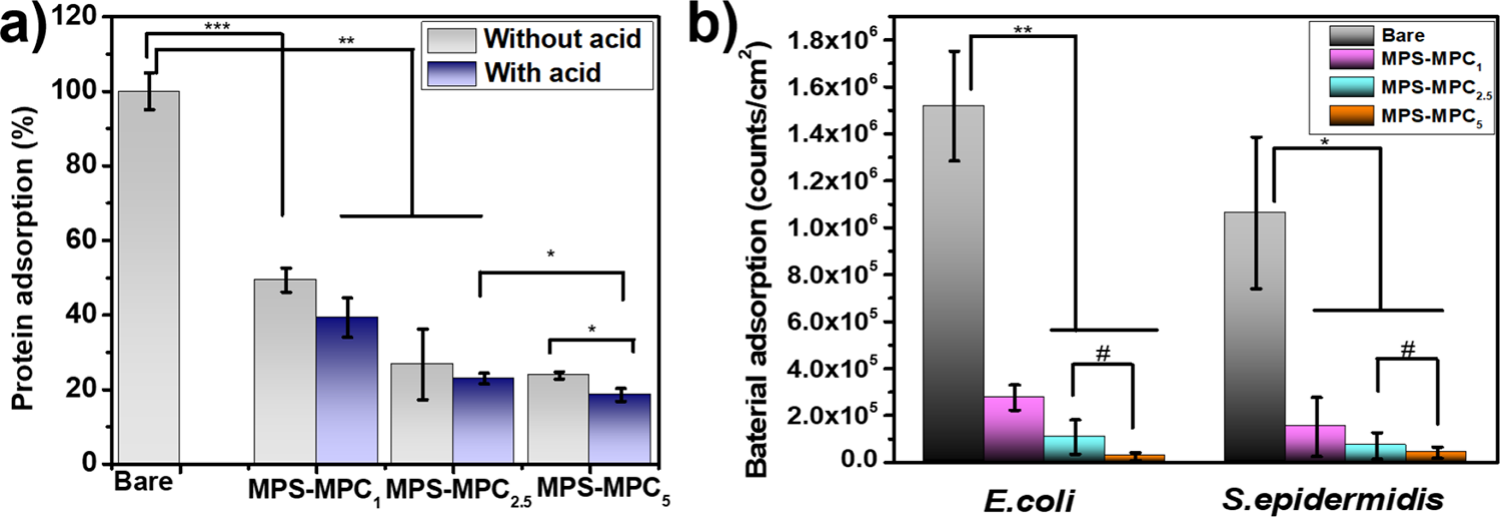
(a) ELISA results of nonspecific BSA absorption on MPS–MPC_*n*_-modified substrates compared with bare wafer, recorded at OD 450 nm using an HRP-labeled tracer (*n* = 3, #*p* > 0.05, *p ≤ 0.5, **p ≤ 0.01, ***p < 0.001). (b) The bacterial quantification of *S. epidermidis* and *E. coli* seized on the bare substrate and zwitterionic oligomeric silatrane films (*n* = 3, #*p* > 0.05, *p ≤ 0.05, **p ≤ 0.01, ***p < 0.001).

Biofouling absorption refers to the adhesion of undesirable materials (such as cells, proteins, and bacteria) from heterogeneous biofluids to submerged surfaces of biosensors through nonspecific interactions. Excessive biofouling can lead to negative consequences, such as increased background signals, the spread of invasive species, and false positive data.^40,41^ First, the ELISA technique was utilized to evaluate the antifouling performance of surfaces modified with zwitterionic oligomeric silatranes, using BSA (one of the most common proteins found in body fluids) as a model foulant to simulate organic fouling. Figure 2a shows the protein resistance levels of the hydration layers formed by zwitterionic polymers MPS–MPC_*n*_ with different degrees of polymerization (DP). After deposition, the protein adsorption on modified wafers was significantly reduced compared to that on bare wafers. Specifically, MPS–MPC_1_, MPS–MPC_2.5_, and MPS–MPC_5_ were able to repel 61, 77, and 81% of protein adhesion, respectively. This finding reveals a correlation between the degree of thiol–ene polymerization and protein resistance levels, which aligns with the previously mentioned concept that better hydrophilic surfaces more effectively prevent the fouling process. To explain this, the N(CH_3_)_4_^+^ ion in phosphatidylcholine of the MPC structure can weakly bind to water molecules in both the primary and secondary hydration layers, enhancing the formation of hydrogen bonds between the surrounding water molecules.^42^ Moreover, MPC zwitterionic material possesses strong hydrophilic, biocompatibility, and excellent antifouling properties to repel the foulant.^30,43,44^ An additional finding is that the difference in reducing protein adsorption between the same polymer in the presence and absence of acetic acid ranged from 3 to 10%. Combined with the previous results on contact angle and thickness, it can be concluded that the addition of acetic acid in the coating solution can accelerate the deposition process, forming a completely thin and uniform film on the surface, thereby achieving an optimal antifouling performance.

In addition to proteins, bacterial fouling also occurs on surfaces and biofilms, forming slimy layers of microbial communities. Notably, *Staphylococcus epidermidis* (*S. epidermidis*) and *Escherichia coli* (*E. coli*) account for a high proportion of medical infections, particularly those related to implanted devices, causing serious issues such as prosthetic heart valve infections, catheter biofilm formation, and bloodstream infections.^45,46^ Moreover, various bacteria in clinical samples can also adhere to biosensor surfaces, leading to reduced sensitivity and accuracy of analytical devices.^47^ In this study, MPS–MPC_*n*_-modified substrates were exposed to *E. coli* (Gram-negative bacteria) and *S. epidermidis* (Gram-positive bacteria) solutions under physiological conditions to investigate their bacterial prevention capabilities. The results, after statistical analysis using ImageJ software, are plotted in Figure 2b, while the fluorescent-stained surfaces were captured by fluorescence microscopy and are visually presented in Figure S8. Compared to the bare surfaces, the number of both types of bacteria observed on the MPS–MPC_*n*_ coatings was significantly reduced, indicating strong hydration layers with effective bacterial repulsion compared to the bare Si wafer. Specifically, *E. coli* and *S. epidermidis* attachment to the MPS–MPC_1_ film was reduced to only 18.2 and 14.3%, respectively, relative to 100% on the untreated surface. Similarly, the percentage of fouling bacteria on the MPS–MPC_2.5_ film decreased to approximately 7.2 and 6.7% for *E. coli* and *S. epidermidis*, respectively. Notably, the MPS–MPC_5_ film offered the best antifouling properties, repelling 98% of *E. coli* and 96% of *S. epidermidis*, which aligns with the protein adsorption results. In general, the antifouling ability of the MPS–MPC_*n*_-modified surface is proportional to the MPC ratio and the coating thickness.

### Modification and Characterization of Single and Mixed Oligomeric Silatranes on Si Wafers

Among all of the films, MPS–MPC_2.5_, with a high conversion rate of 90.5% for thiol–ene polymerization, performed excellently in both bacterial and protein prevention. Notably, when treated with acetic acid in the coating solution, MPS–MPC_2.5_ resisted approximately 90% of bacterial adhesion and nearly 80% of protein adsorption. Meanwhile, MPS–MPC_5_ also exhibited superior antifouling properties, even with a thicker layer and a degree of polymerization of 8.31. The general principle for designing a sensor surface with mixed coatings involves using an extended component with exposed functional groups to immobilize biorecognition molecules and a short component, serving as a passivative material. This approach ensures effective detection, creating a high-selectivity biosensor that minimizes false positive or false negative results.^48,49^ The strategy inspired the combination of the two functional components in this research for the construction of functionalized surfaces that can be applied to developing biointerfaces. In the mixed oligomeric coating, MPS–MPC_2.5_ is preferred for providing splendid surface hydrophilicity, fouling resistance, and high packing density, while MPS–PEGMACOOH_*m*_ with the functional carboxylate group plays a critical role in postmodification as well as further supporting the antifouling ability of the mixed coating. The three coating mixtures used in the following experiments are described in Table 2.

**Table 2:** Detailed Components of Polymers Used in Coating Mixtures

**sample name**	**coating solution formula**
Mix 1	3 mL of MPS–PEGMACOOH_2.5_ + 3 mL of MPS–MPC_2.5_ (molar fraction: 5:5)
Mix 2	3 mL of MPS–PEGMACOOH_2.5_ + 3 mL of MPS–MPC_2.5_ (molar fraction: 7:3)
Mix 3	3 mL of MPS–PEGMACOOH_5_ + 3 mL of MPS–MPC_2.5_ (molar fraction: 5:5)

**Figure 3:**
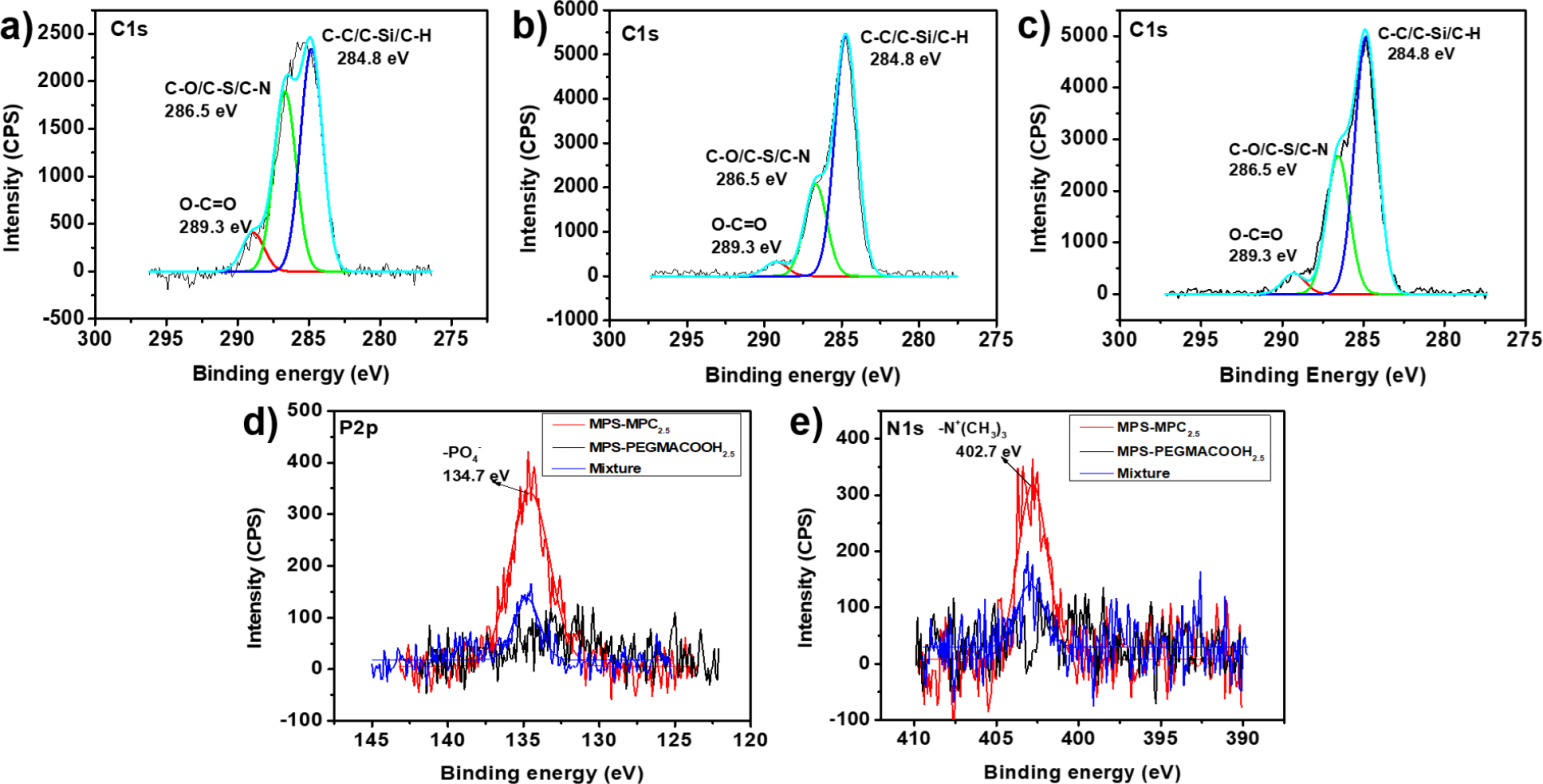
C 1s XPS spectra of coated Si wafers with (a) MPS–MPC_2.5_, (b) MPS–PEGMACOOH_2.5_, and (c) Mix 2. (d) P 2p and (e) N 1s XPS spectra of MPS–MPC_2.5_, MPS–PEGMACOOH_2.5_, and Mix 2-coated Si wafers.

Initially, X-ray photoelectron spectroscopy (XPS) was used to confirm the presence of MPS–MPC_*n*_ and MPS–PEGMACOOH_*m*_ in both single and mixed coatings on the Si wafers after modification by analyzing the surface element composition and chemical state. Figure 3 illustrates the high-resolution XPS spectra of C 1s, N 1s, and P 2p for substrates modified with MPS–MPC_2.5_, MPS–PEGMACOOH_2.5_, and Mix 2. In the C 1s spectra, as shown in Figure 3a–c, the signal at the binding energy (BE) of 289.3 eV corresponds to the methacrylate group present in all molecular structures of MPS–MPC_2.5_, MPS–PEGMACOOH_2.5_, and Mix 2. Additionally, the C–O/C–S/C–N bonds in the two zwitterionic polymers and C–C/C–Si/C–H bonds in the silatrane structure were observed at BEs of 286.5 and 284.8 eV, respectively, indicating successful surface silanization. When comparing Figure 3b,c, the intensity of the saturated hydrocarbon peak at 284.8 eV in the MPS–PEGMACOOH_2.5_ and Mix 2 coatings is higher than that in MPS–MPC_2.5_, due to the abundance of carboxylated PEGMA branches on the oligomeric silatrane.^50^Figure 3d shows the P 2p spectra of modified samples, where the presence of MPS–MPC_2.5_ in both individual and mixed coatings is confirmed by the peak of −PO_4_^–^ in the MPC at 134.7 eV, which is absent in the MPS–PEGMACOOH2.5 spectrum. The N 1s spectrum, revealed in Figure 3e, shows a peak at 402.7 eV corresponding to the quaternary amine −N^+^(CH_3_)_3_ in MPS–MPC_2.5_ and mixed oligomeric silatrane, a peak not present in the MPS–PEGMACOOH_2.5_-modified sample.^50,51^ The higher intensity of signal in MPS–PEGMACOOH_2.5_ and Mix 2 spectra compared to the MPS–MPC_2.5_ one can be attributed to higher content of C element due to the intrinsic structure, which will be further reflected by the following discussed coating thickness. Notably, the ratio between the highest intensity of −PO_4_^–^ and COO^–^ representative peaks in the spectrum of a single MPS–MPC_2.5_ coating is 1.17, approximate to the theoretical ratio of 1. This value dramatically drops to 0.34 in Mix 2 coating, showing a nearly four-time increase in the proportion of COO^–^ in the coating with a combination of both MPS–MPC_2.5_ and MPS–PEGMACOOH_2.5_. Such a significant change can be ascribed to the presence of PEGMACOOH with three COO^–^ groups in each molecular structure, evidencing that both polymers have been successfully deposited on the surface. Moreover, the element composition percentages of the tested samples are given in Table S1. The marked reduction in the P element composition percentage from 3.42% in the MPS–MPC_2.5_ film to 1.58% in the Mix 2 film further supports the conclusion that both polymers were effectively deposited. In conclusion, the XPS data demonstrated that the deposition of oligomeric silatranes on Si wafers was successfully accomplished. In addition to the evidence from XPS, the water contact angles and antifouling behavior further demonstrate that the coverage density of the oligomers was virtually complete. Notably, the water contact angles of the oligomer-coated surfaces were significantly reduced compared to bare surfaces, particularly those containing the MPC component (Figure 1). Moreover, protein and bacterial adhesion to the zwitterionic coatings was dramatically reduced compared to bare surfaces, with the oligomer coatings decreasing bacterial adhesion by more than 90% (Figure 2 and Figure S8). Therefore, it is reasonable to conclude that the surfaces were thoroughly covered with the oligomers.

**Figure 4:**
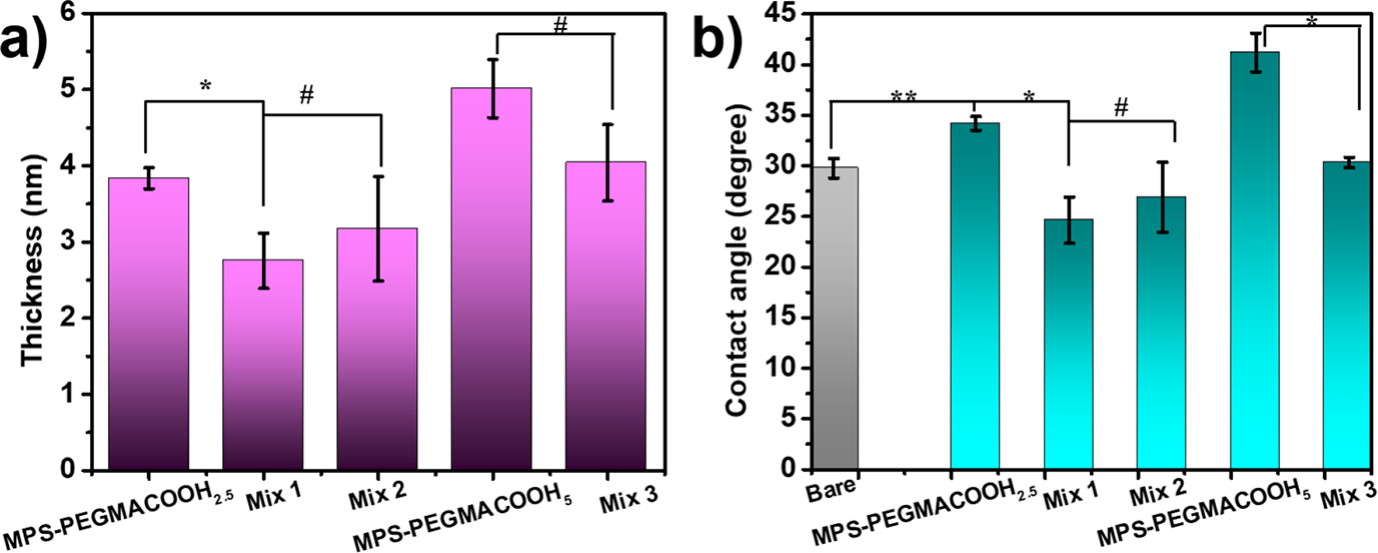
(a) Thicknesses and (b) static water contact angles of MPS–PEGMA_*m*_ and mixed oligomeric coatings (*n* = 3, #*p* > 0.05, *p ≤ 0.5, **p ≤ 0.01, ***p < 0.001).

Subsequently, the thickness and wettability of the polymer coatings containing carboxylated PEGMA were measured and recorded using an ellipsometer and a goniometer, respectively. All coating solutions were prepared in anhydrous methanol at a final concentration of 5 mM for MPS–PEGMACOOH_2.5_, MPS–PEGMACOOH_5_, and the three mixtures described in Table 2. The thicknesses of the PEGMACOOH-containing oligomeric silatrane films were determined using an ellipsometer and are illustrated in Figure 4a. The first observation is that MPS–PEGMACOOH_*m*_, contributed by the long backbone of carboxylated PEGMA, forms a layer that is thicker than that previously recorded for MPS–MPC_*n*_ (Figure 1a). Among these, MPS–PEGMACOOH_5_ forms the thickest layer (5.01 ± 0.38 nm) compared to those of the polymer mixtures and MPS–PEGMACOOH_2.5_. Notably, all mixed films have lower thicknesses compared to the single MPS–PEGMACOOH_*m*_, with the thicknesses of Mix 1, Mix 2, and Mix 3 coatings being 3.17 ± 0.36, 3.17 ± 0.69, and 4.04 ± 0.50 nm, respectively. Additionally, the element compositions from XPS analysis shown in Table S1 suggest that an increase in the C 1s proportion correlates with the enhanced thickness of the oligomeric silatrane coatings. Another aspect to mention, against MPS–MPC_*n*_ polymers with the same polymerization ratio, it can be seen that MPS–PEGMACOOH_*m*_ polymers (Figure 1) have thicker coating. This is attributed to the longer molecular structure of the PEGMACOOH monomer than that of MPS–MPC_*n*_, which also facilitates the exposure of COOH functional groups on the antifouling surface for further conjugation. Likewise, mixed coatings of these two polymers expressed thicknesses that fell between those of the individual coatings. The water contact angles (WCAs) were then recorded to assess the hydrophilicity of the modified samples, as shown in Figure 4b. As expected, MPS–PEGMACOOH_2.5_ and MPS–PEGMACOOH_5_ exhibited hydrophilic surfaces with WCAs of 34.2 ± 0.7 and 41.1 ± 1.9°, respectively. This hydrophilicity is not only due to the functional COOH group but also thanks to the polyethylene glycol backbone structure in MPS–PEGMACOOH_*m*_. However, the complexity and bulkiness of the polymeric structures might account for the slightly higher contact angles observed for these polymer coatings compared to simple COOH-terminated layers.^52−54^ When combined with the zwitterionic MPS–MPC_*n*_ polymer in different molar fractions, the WCA values for Mix 1, Mix 2, and Mix 3 significantly decreased to 24.6 ± 2.3, 26.9 ± 3.5, and 30.3 ± 0.5°, respectively. Therefore, mixing oligomeric silatranes has proven to be a promising strategy for surface functionalization, leading to the formation of a thin film with strong antifouling properties.

### Molecular Detection on Mixed Oligomeric Silatrane Coatings

**Figure 5:**
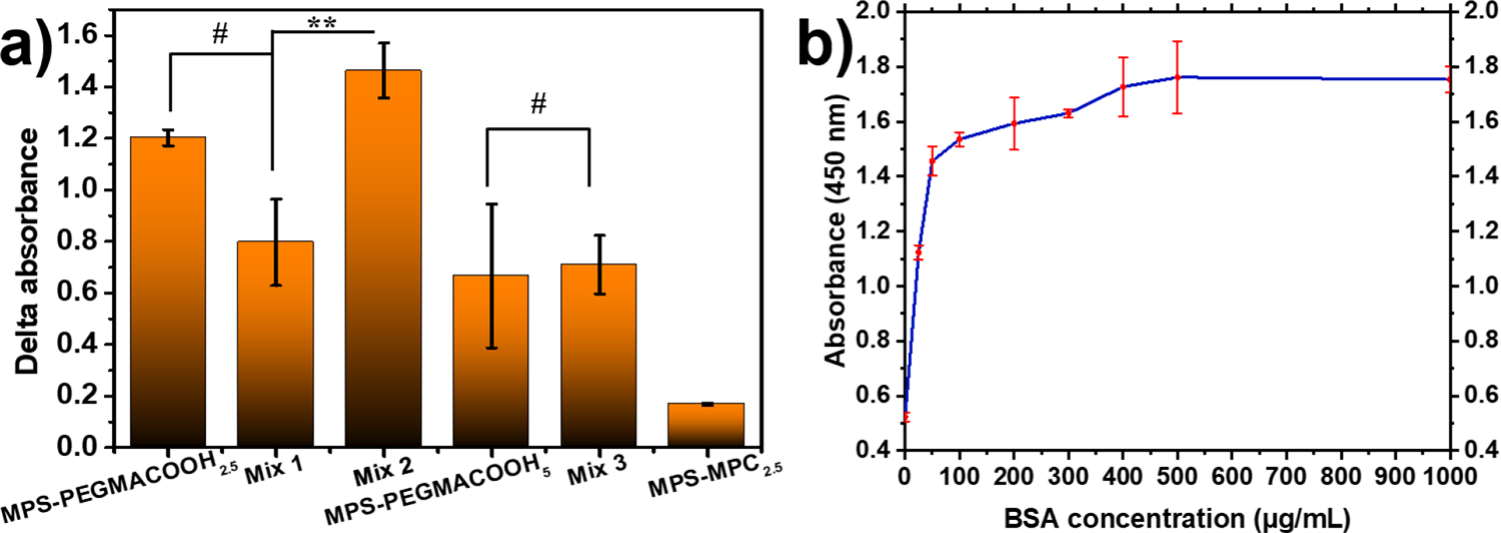
(a) ELISA delta absorbances reflect BSA immobilization on modified substrates. (b) ELISA absorbance-resolved graph of the Mix 2 film in different BSA concentrations.

Albumin is the most common protein in bodily circulating fluids, playing essential roles in maintaining osmotic pressure, transporting various molecules such as hormones and fatty acids, and regulating the pH. It is a multifunctional protein produced primarily by the liver and found in high concentrations in blood plasma and serum. Changes in albumin levels can be indicative of various diseases and health conditions, particularly liver and kidney diseases.^55−57^ Bovine serum albumin (BSA) is a suitable model protein for in vitro studies.^58^ In addition, BSA is commercially available, highly stable, and water-soluble with an isoelectric point (IEP) at pH 4.5–5.0, making it negatively charged at neutral pH.^34^ Therefore, BSA was chosen to investigate the application potential of polymeric coatings in the postmodification of biomolecules via EDC/NHS amine coupling chemistry. The study began by exploring the dependence of BSA immobilization via covalent bonding under three pH conditions: pH 2 (positive charge), pH 5 (neutral charge), and pH 7.5 (negative charge) (Figure S9). Figure 5 presents the experimental results where BSA was detected through indirect ELISA. The amount of BSA captured on the modified mixed oligomeric silatrane by activating terminal carboxylic acid with EDC/NHS was compared to that of the control groups. Figure 5a discloses the delta absorbance (ΔAbs) of BSA detection on modified samples after the background signal was subtracted. Accordingly, Mix 2, containing 70% MPS–PEGMACOOH_2.5_ with 30% MPS–MPC_2.5_ in molar fraction, exhibited the highest signal of BSA immobilization compared with the other modified samples. On the one hand, the high DP and bulky structure of MPS–PEGMACOOH_5_ might cause entanglement, hindering BSA access to the COOH groups, leading to weak absorbance in both single MPS–PEGMACOOH_5_ and Mix 3 coatings. On the other hand, Mix 1, with the lowest concentration of PEGMACOOH, also absorbed only a small amount of BSA, likely due to the lack of available COOH end groups. Consequently, Mix 2, which combines MPS–PEGMACOOH_2.5_ with MPS–MPC_2.5_ in a 7:3 molar fraction, is an excellent candidate for the surface modification of biosensors. This mixture holds dual functionality: it strongly prevents nonspecific absorption due to the zwitterionic MPC and hydrophilic PEG, and it also specifically captures proteins due to the terminal carboxyl groups.^23^ Mix 2 was further applied to capture BSA in concentrations ranging from 25 to 1000 μg/mL, as shown in Figure 5b. The figure demonstrates the relationship between BSA concentration and signal intensity, highlighting the nonlinear response between these factors. The binding capability of BSA on the Mix 2-modified surface, facilitated by the activation of carboxylic acid with EDC/NHS, is demonstrated by an initially rapid increase in signal as the BSA concentration rises from 0 to 25 μg/mL. Subsequently, the rate of increase gradually slows until it eventually stabilizes at a concentration of 400 μg/mL, indicating the saturation of BSA. A linear relationship between BSA concentration and detected absorbance is observed in the range of 0–50 μg/mL. The lowest BSA concentration tested, 25 μg/mL, is significantly lower than the typical serum BSA concentration (35–45 mg/mL) and even lower than that of some other serum proteins. This suggests that our interface is capable of detecting both physiological and pathological thresholds of target proteins.

**Figure 6:**
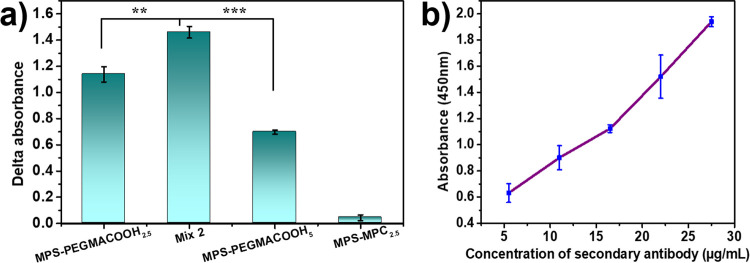
(a) ELISA delta absorbances reflect primary Ab secured on modified substrates. (b) ELISA absorbance-resolved graph of Mix 2 film in different concentrations of the HRP-conjugated secondary antibody.

This work aimed to evaluate the conjugation between terminal COOH groups on mixed oligomeric silatrane coatings and anti-BSA IgG as the primary antibody via EDC/NHS coupling. This conjugation serves as a bioreceptor for detecting the secondary antibody of horseradish peroxidase (HRP)-conjugated goat antirabbit IgG Ab as the targeted analyte. The detection mechanism relies on the labeled HRP enzyme catalyzing a colorimetric reaction with the TMB substrate, producing a stopped yellow color that can be measured at a wavelength of 450 nm.^59^ Depicted in Figure 6a is the difference in absorption signals with and without the conjugated anti-BSA IgG primary antibody, which confirms that this antibody was successfully attached to the carboxylated PEGMA active site, allowing for the subsequent attachment of the secondary antibody. Among all samples, Mix 2 once again manifested the greatest absorbance intensity of the analyte along with the smallest background signal, resulting in the highest signal difference after subtraction. Thus, Mix 2, which combines MPS–PEGMACOOH_2.5_ with MPS–MPC_2.5_ in a 7:3 molar ratio, secures the best performance in both countering nonspecific adsorption and enhancing target detection. Another point worth discussing is the lack of correlation between the coating thickness and its ability to capture BSA, whether the coating consisted of a single MPS–PEGMACOOH_*m*_ layer or a mixed MPS–PEGMACOOH_2.5_ and MPS–MPC_2.5_ layer. Figure 6b presents the correlation between the HRP-conjugated secondary antibody concentration and the signal obtained when the Mix 2 coating and a constant concentration of analyte were applied. In the testing range of 5.5–27.5 μg/mL, a nearly linear relationship was observed between the concentration of the detecting agent and the absorbed signal. Therefore, it can be assumed that the primary antibody was consistently immobilized on the Mix 2-modified surface with proper orientation, ensuring accurate detection of the secondary antibody.

## Conclusions

This work presents the development of mixed oligomeric silatranes for creating dual-functional biointerfaces suitable for a wide range of biological sensing applications. The oligomeric silatranes, MPS–MPC_*n*_ and MPS–PEGMACOOH_*m*_, were synthesized using thiol–ene photopolymerization and subsequently deposited on Si wafers, resulting in thin, uniform films. By adjusting the composition and conditions of the coating solutions, we achieved the desired features that were achieved. Notably, films formed from MPS–MPC_*n*_ solutions exhibited high hydrophilicity and effectively resisted bacterial adhesion and protein absorption. In mixed solutions, a coating consisting of 70% MPS–PEGMACOOH_2.5_ and 30% MPS–MPC_2.5_ yielded the thinnest layer and exhibited the highest wettability among the mixtures tested. Furthermore, these mixed oligomeric silatranes successfully established a platform for BSA antigen and antibody detection using the ELISA technique. Remarkably, the mixture of 70% MPS–PEGMACOOH_2.5_ and 30% MPS–MPC_2.5_ demonstrated superior performance by minimizing nonspecific signals and achieving the highest analyte absorbance. Thus, the mixed oligomeric silatrane offers an ideal platform for biosensor development, combining excellent antifouling properties from the zwitterionic oligomer MPS–MPC_2.5_ with the biomolecule immobilization capabilities of MPS–PEGMACOOH_2.5_ through EDC/NHS coupling. This approach is anticipated to become a powerful strategy for diversifying biointerfaces with different functions, particularly for biosensor applications.

## Supporting Information Available

^1^H NMR spectrum of carboxylated PEGMA and PEGMAOH; ^13^C NMR spectrum of Carboxylated PEGMA; ATR-FTIR spectra of carboxylated PEGMA and PEGMAOH; representative ^1^H NMR spectra of MPS, MPS–MPC_*n*_, and MPS–PEGMACOOH_*m*_; ^1^H NMR spectra of MPS–MPC_*n*_ with different ratios; ^1^H NMR spectra of MPS–PEGMACOOH_*m*_ with different feed ratios; images of water droplets on the surface of MPS–MPC_*n*_-modified samples; XPS-based analysis in surface element composition of bare and modified Si wafers; visually fluorescent images of bacteria *S. epidermidis* and *E. coli* adsorbed on the bare substrate and zwitterionic oligomeric silatrane films; the influence of different pH to the immobilization of BSA on surface coated with MPS–PEGMACOOH_*m*_ (PDF)
