# Supplementary material for: Development of Functional Biointerface Using Mixed Zwitterionic Silatranes
Source: Langmuir. 2024 Nov 11;40(46):24516–27. doi: 10.1021/acs.langmuir.4c03302 (PMC11580372; doi:10.1021/acs.langmuir.4c03302)
Supplement: Supplementary file 1 — la4c03302_si_001.pdf [file la4c03302_si_001.pdf]

SUPPORTING INFORMATION

Development of Functional Biointerface Using Mixed Zwitterionic Silatranes

Thi Anh Hong Tran [a,b], *Van Truc Vu* [b], *Chun-Jen Huang* [b, c,] *

[a] Department of Biomedical Sciences and Engineering, National Central University, Jhong-Li, Taoyuan 320, Taiwan.

[b] Department of Chemical & Materials Engineering, National Central University, Jhong-Li, Taoyuan 320, Taiwan.

[c] School of Materials Science and Engineering, The University of New South Wales, Sydney, NSW 2052, Australia.

* Corresponding author. Email: cjhuang@ncu.edu.tw (C.-J. H.)

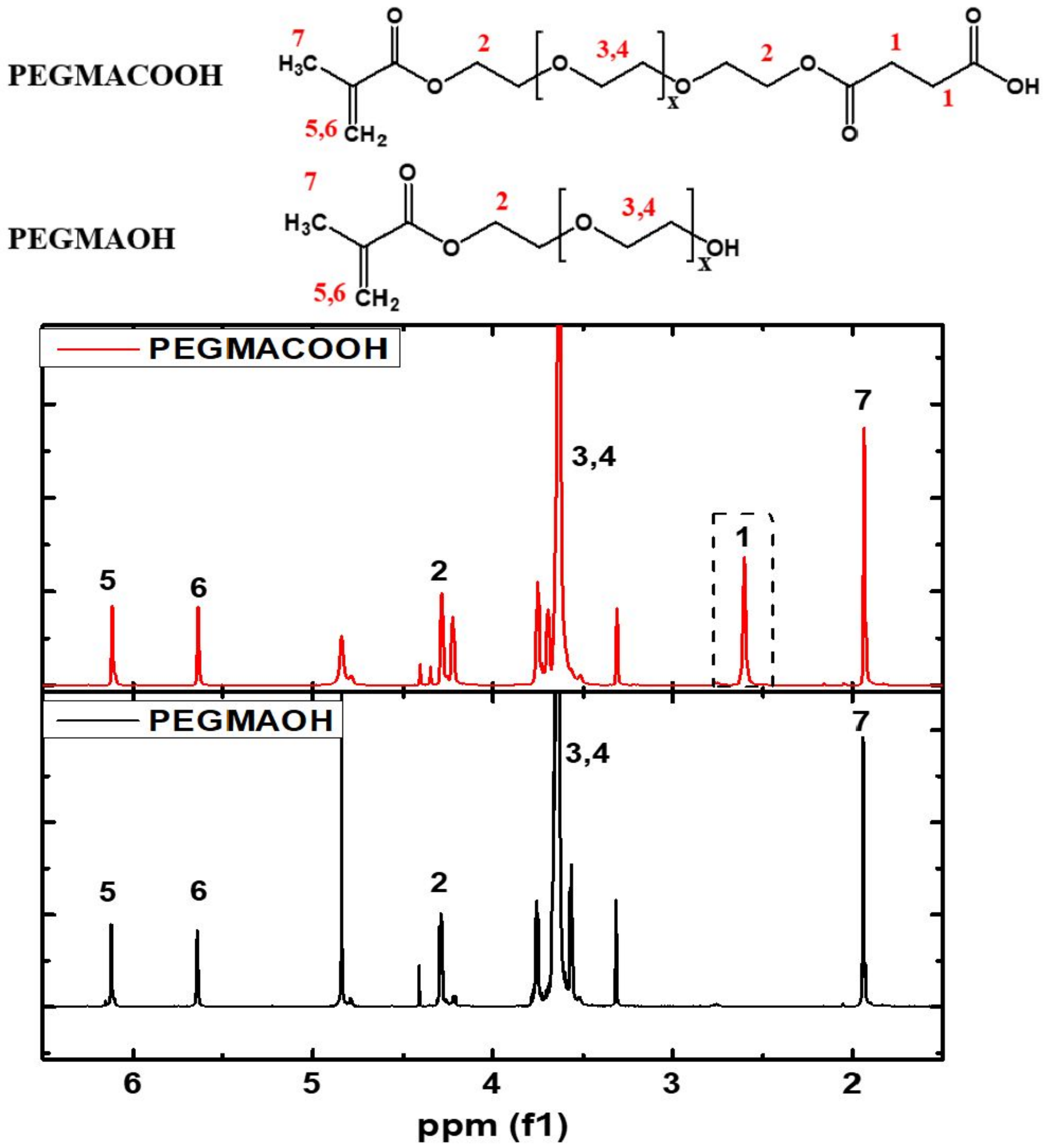

Figure S1. ^{1}H NMR spectra of Carboxylated PEGMA and PEGMAOH.

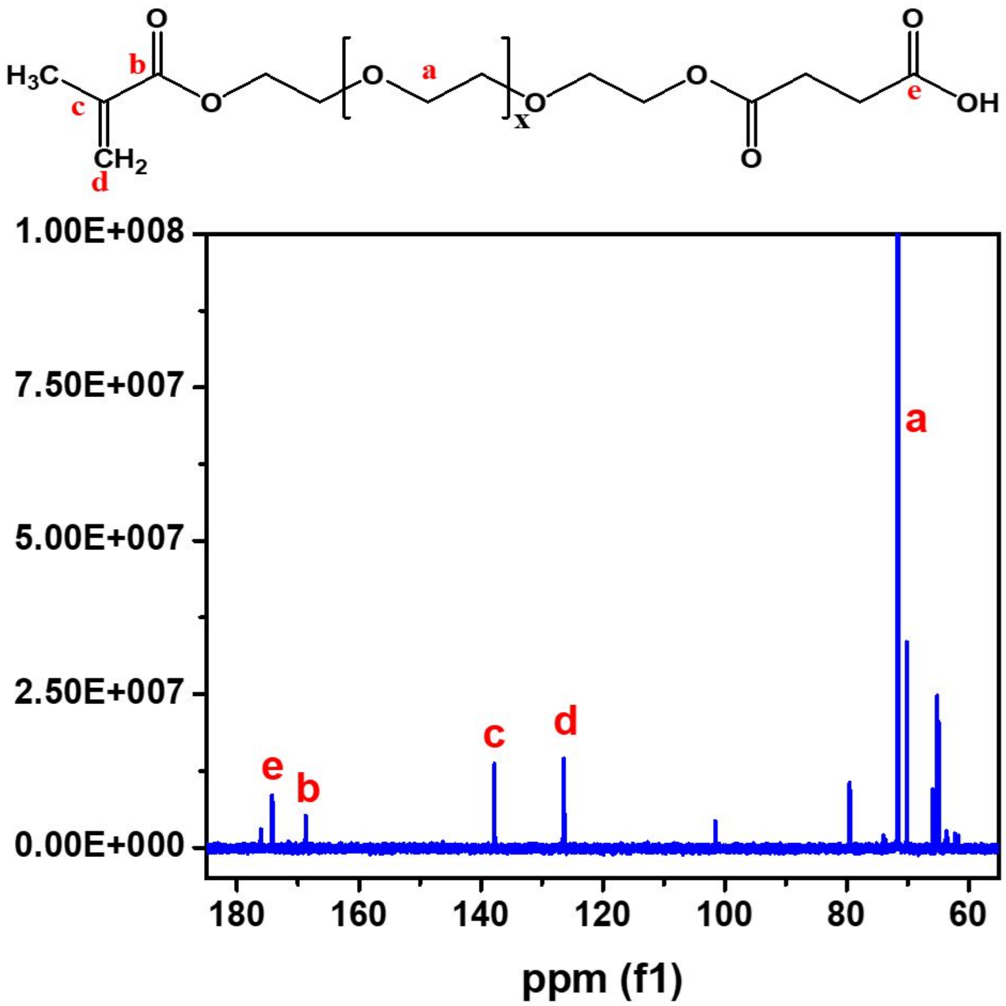

Figure S2. ^{13}C NMR spectrum of Carboxylated PEGMA.

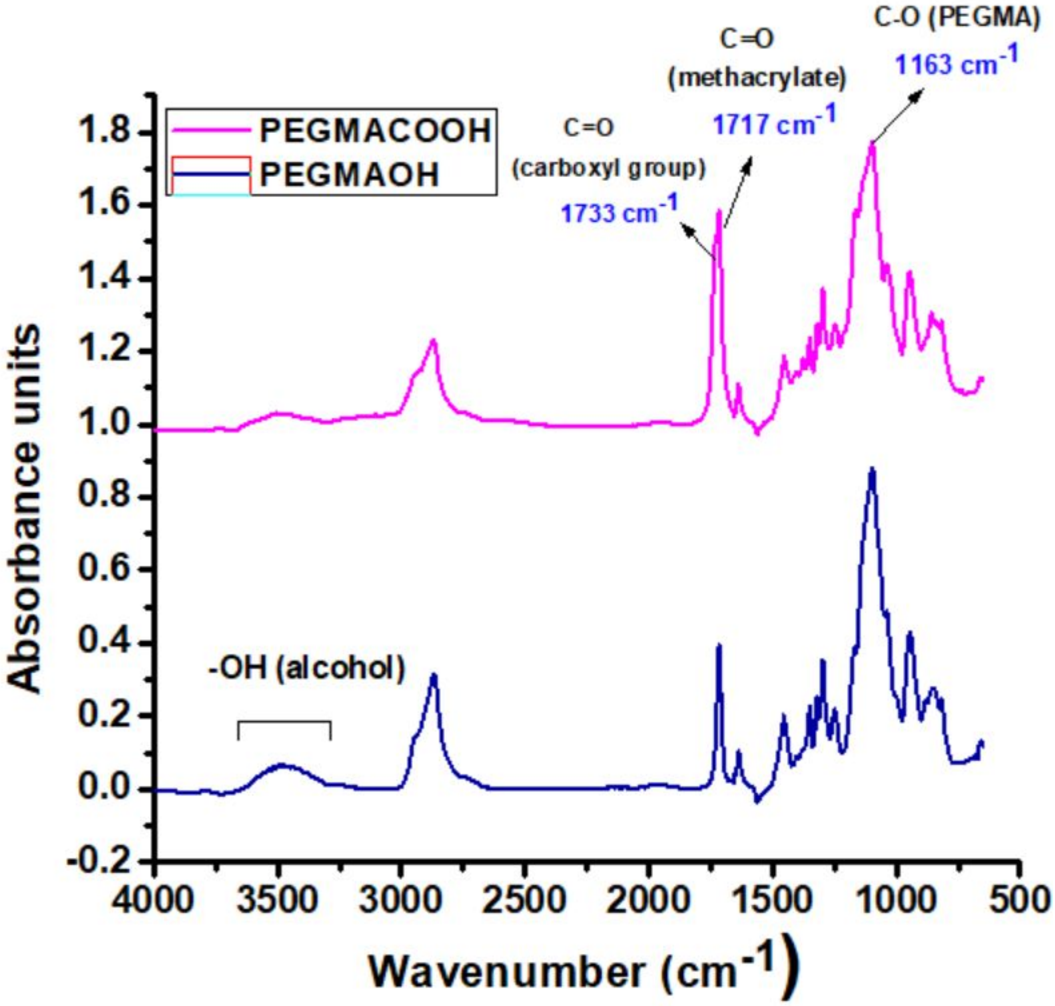

Figure S3. ATR-FTIR spectra of Carboxylated PEGMA and PEGMAOH.

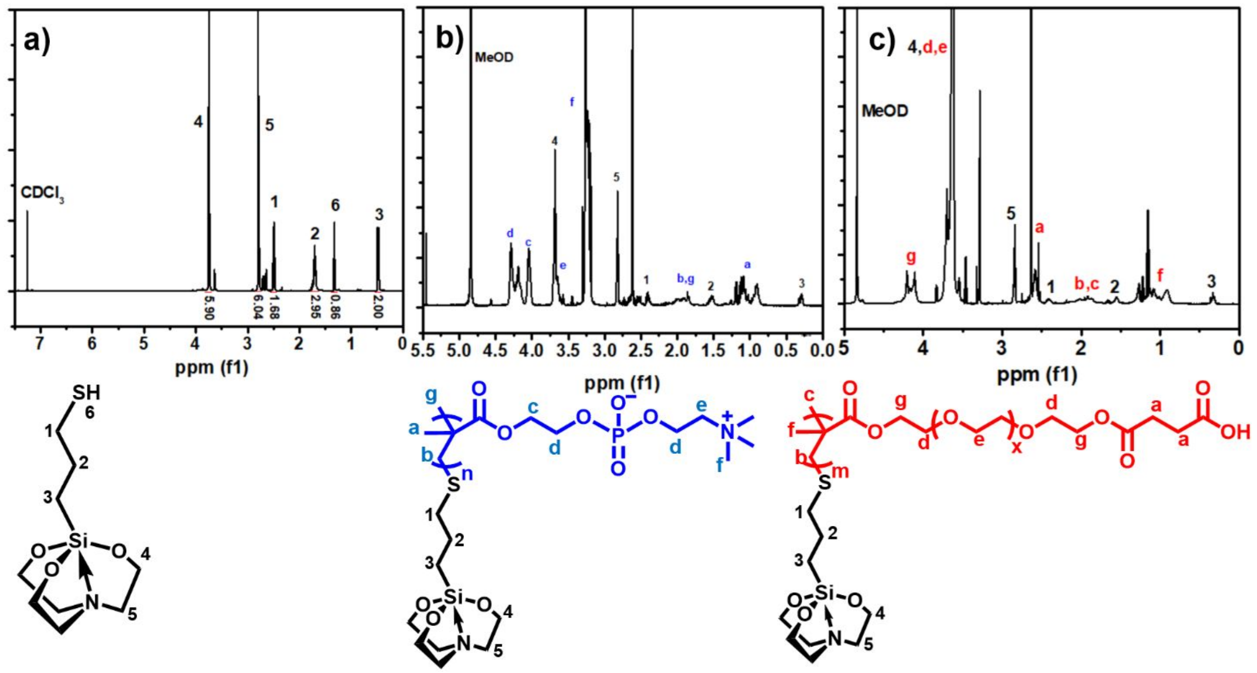

Figure S4. Representative ¹H NMR spectra of (a) MPS in $CDCl_3$, (b) MPS−MPC_n in MeOD, and (c) MPS−$PEGMACOOH_m$ in MeOD.

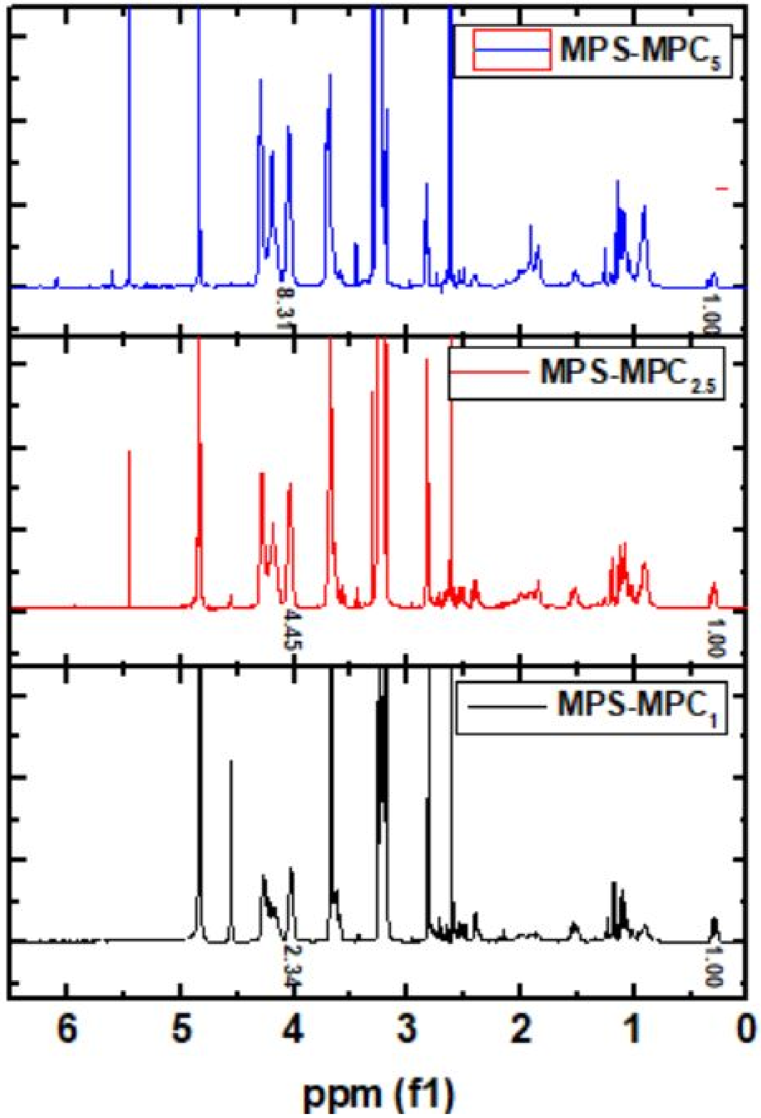

Figure S5. ^{1}H NMR spectra of MPS−MPC$_n$ with different ratios. ^{1}H NMR (MeOD, 600MHz): δ (ppm) = 0.28−0.38 (**3**, Si**CH$_2$**CH$_2$), 0.90−1.35 (**a**, α-CH$_3$), 1.49−1.65 (**2**, SiCH$_2$**CH$_2$**), 1.85−2.28 (**b**,**g**, S**CH$_2$**C, **CH$_2$**C), 2.49−2.66 (**1**,CH$_2$**CH$_2$**S), 2.78−2.94(**5**,N(**CH$_2$**CH$_2$)$_3$), 3.20−3.49 (**f**, CH$_2$N(**CH$_3$)$_3$**), 3.62−3.79 (4,**e**, **CH$_2$**N (CH$_3$)$_3$, O**CH$_2$**CH$_2$), 4.00−4.14 (**c**, O**CH$_2$**), 4.14−4.41 (**d**, **CH$_2$**PO$_4$**CH$_2$**).

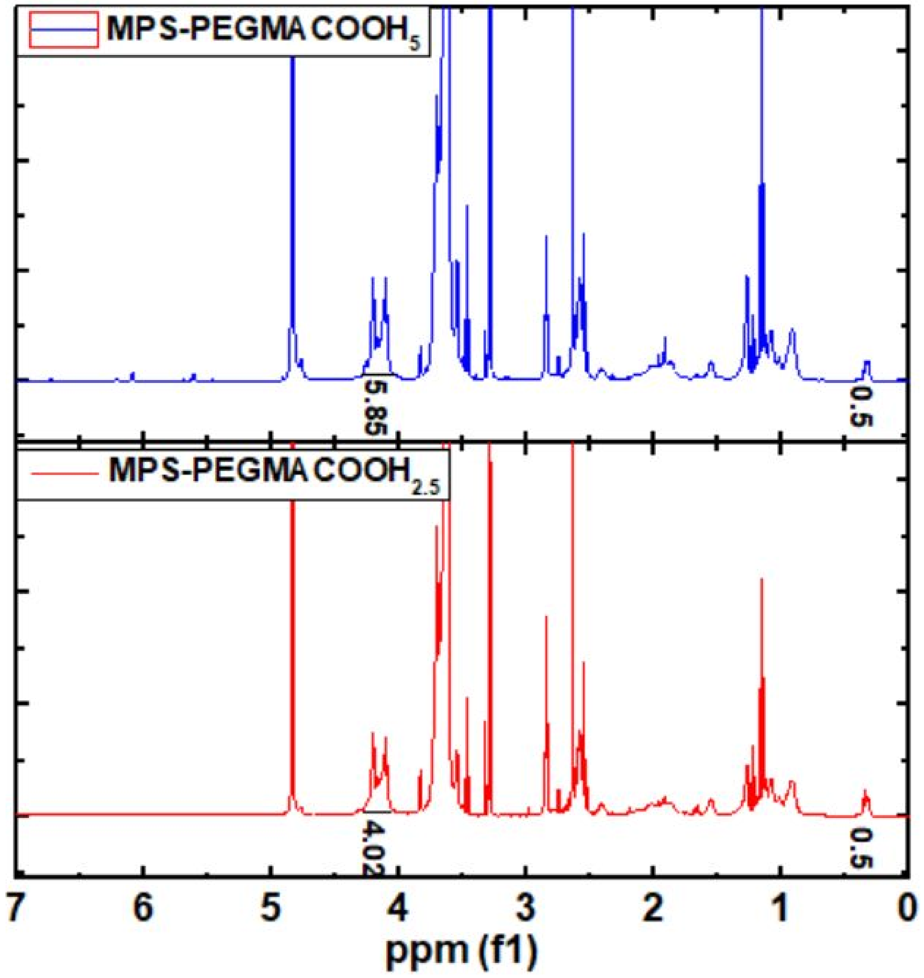

Figure S6. ^{1}H NMR spectra of MPS−PEGMACOOH$_m$ with different feed ratios. ^{1}H NMR (MeOD, 600MHz): δ (ppm) = 0.28−0.38 (**3**, Si**CH$_2$**CH$_2$), 0.90−1.35 (**f**, α-CH$_3$), 1.49−1.65 (**2**, SiCH$_2$**CH$_2$**), 1.85−2.28 (**b,c**, S**CH$_2$**C, **CH$_2$**C), 2.49−2.66 (**1**,CH2**CH$_2$**S), 2.54−2.42 (**a**,(CH2)2COOH), 2.78−2.94 (**5**,N(**CH$_2$**CH$_2$)$_3$), 3.54-3.79 (**4,d,e** O(CH2)2O), SiO**CH$_2$**CH$_2$), 4.10−4.21 (**g**, O(CH2)2O(CO)).

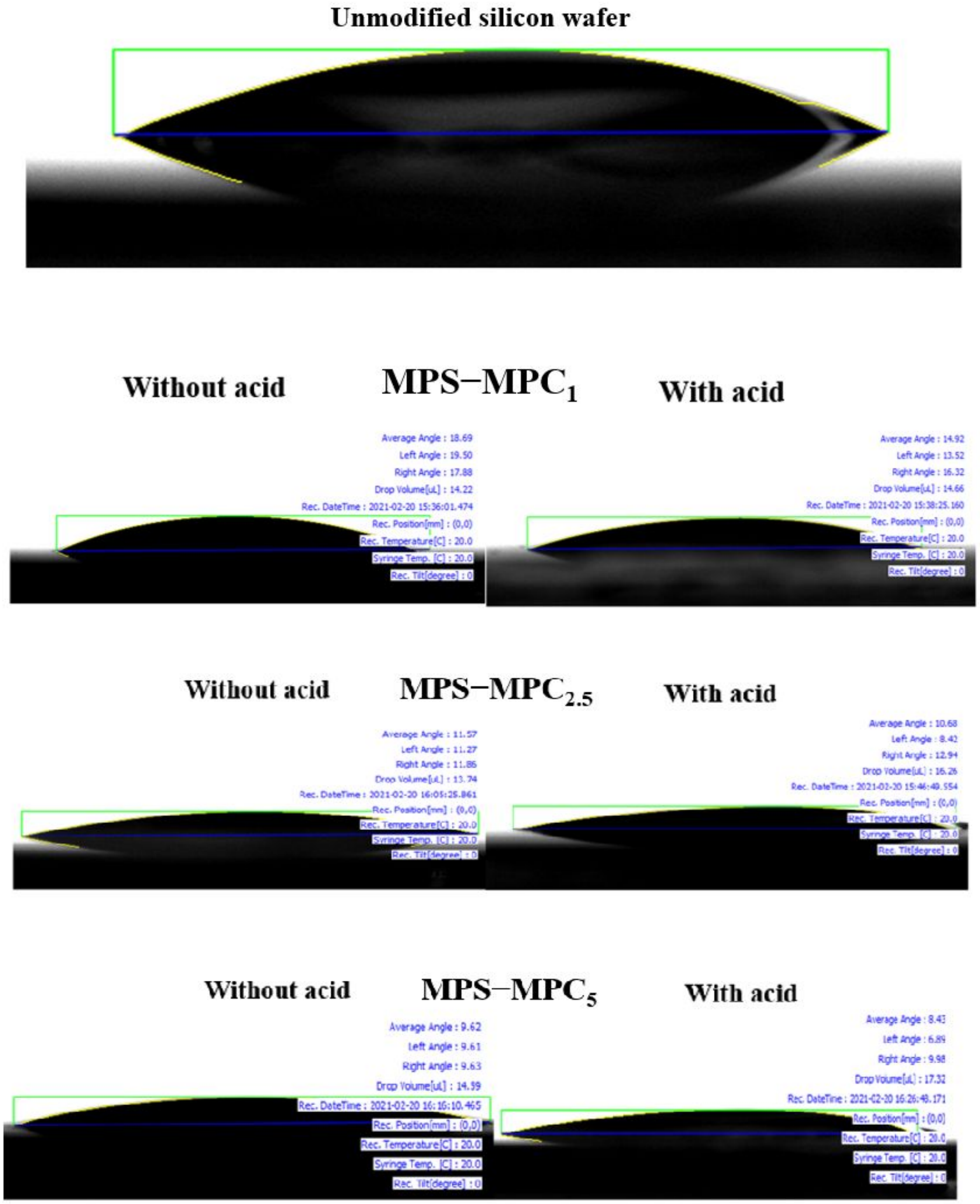

Figure S7. Images of a water droplets on the surface of MPS−MPC$_n$ modified samples.

Table S1: XPS-based analysis in surface elements composition of bare and modified Si wafers.

Sample name	Element composition (%)					
	C1s	N1s	O1s	P2p	Si2p	S2p
Bare	36.8	1.5	32.7	0.1	26.5	2.3
MPS−$MPC_{2.5}$	34.6	2.0	35.5	3.4	23.8	2.2
MPS−$PEGMACOOH_{2.5}$	44.5	1.0	32.7	1.0	18.9	2.1
Mix 2	43.9	1.7	27.3	1.6	21.7	3.8

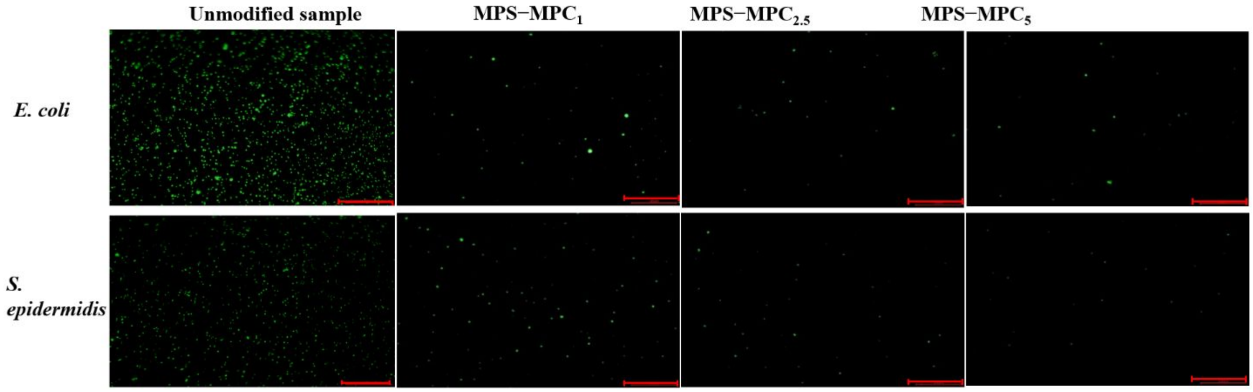

Figure S8. Visually fluorescent images of bacteria *S. epidermis* and *E. coli* adsorbed on the bare substrate and zwitterionic oligomeric silatrane films. The scale bar is 100 μm.

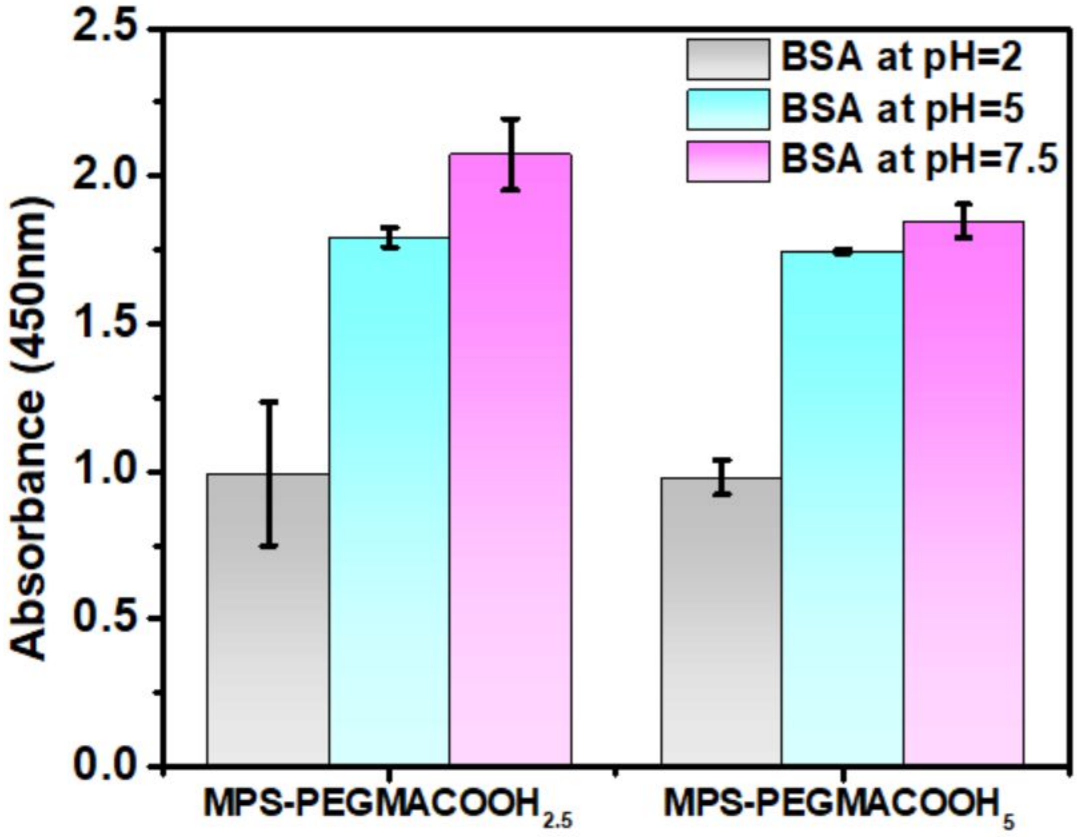

Figure S9. The influence of different pH to the immobilization of BSA on surface coated with MPS−PEGMACOOH$_m$.
